# Chemical characterization and biological activity of six different extracts of propolis through conventional methods and supercritical extraction

**DOI:** 10.1371/journal.pone.0207676

**Published:** 2018-12-04

**Authors:** Danielle Devequi-Nunes, Bruna Aparecida Souza Machado, Gabriele de Abreu Barreto, Jéssica Rebouças Silva, Danielle Figuerêdo da Silva, José Luiz Carneiro da Rocha, Hugo Neves Brandão, Valéria M. Borges, Marcelo Andres Umsza-Guez

**Affiliations:** 1 SENAI CIMATEC University Center, Health Institute of Technologies (ITS CIMATEC), National Service of Industrial Learning–SENAI, Salvador, Bahia, Brazil; 2 Gonçalo Moniz Institute, Oswaldo Cruz Foundation, Salvador, Bahia, Brazil; 3 Federal University of Bahia, Salvador, Bahia, Brazil; 4 Estadual University of Feira de Santana, Feira de Santana, Bahia, Brazil; College of Agricultural Sciences, UNITED STATES

## Abstract

Propolis is a natural product with many demonstrated biological activities and propolis extract has been used in the food, pharmaceutical and cosmetics industries. Different works have showed the variations in the chemical composition, and consequently, on the biological activity of the propolis that are associated with its type and geographic origin. Due to this study evaluated propolis extracts obtained through supercritical extraction and ethanolic extraction (conventional) in three samples of different types of propolis (red, green and brown), collected from different regions in Brazil (state of Bahia). Analyses were performed to determine the humidity, water activity, the content of total ash, proteins, lipids and fiber in raw propolis samples. The content of phenolic compounds, flavonoids, *in vitro* antioxidant activity (DPPH), catechin, ferulic acid and luteolin and antimicrobial activity against two bacteria (*Staphylococcus aureus* and *Escherichia coli*) were determined for all extracts. For the green and red ethanolic extracts the anti-leishmanicidal potential was also evaluated. The physicochemical profiles showed agreement in relation to the literature. The results identified significant differences among the extracts (p>0.05), which are in conformity with their extraction method, as well as with type and botanical origin of the samples. The extraction with supercritical fluid was not efficient to obtain extracts with the highest contents of antioxidants compounds, when compared with the ethanolic extracts. The best results were shown for the extracts obtained through the conventional extraction method (ethanolic) indicating a higher selectivity for the extraction of antioxidants compounds. The red variety showed the largest biological potential, which included the content of antioxidants compounds. The results found in this study confirm the influence of the type of the raw material on the composition and characteristics of the extracts. The parameters analysis were important to characterize and evaluate the quality of the different Brazilian propolis extracts based on the increased use of propolis by the natural products industry.

## Introduction

Propolis is a resinous material produced by bees (*Apis melífera L*.), primarily from plants, as a sticky exudate from leaf and flower buds, shoots, stems and fruit [1]. In Brazilian territory, 13 different types of propolis have been categorized based on physical-chemical characteristics [2–4]. *Baccharis dracunculifolia* is a type of common plant that produces green propolis, which is rich in compounds with prenylated phenylpropanoids, triterpenoids, benzoic acid and chlorogenics [5]. The *Dalbergia ecastophyllum (L*.*) Taub*. *(Fabaceae)* species are mangrove natives used by bees to produce the red propolis. Brown propolis is produced by the *Copaifera* species and mainly contains flavonoids and terpenes [6–8].

Bees use propolis to fill gaps and narrow spaces in their hives and to prevent microbial contamination in the egg deposition nests and respiratory outlets. The antimicrobial properties of propolis are important for maintaining a healthy hive environment for the bee colony [9].

In terms of chemical composition, propolis is generally composed of 50% resin, 30% wax, 10% essential oils, 5% pollen, and 5% other substances, including the derivatives of cinnamic acid, phenolic acids, substituted benzoic acids, amino acids and flavonoids [10–11]. The biological effects and chemical composition of propolis depend on various factors, such as the types of vegetable sources, the geographic origin, the season of the year and the time of collection [12–13].

Ethanol has been the most used solvent to obtain low‐wax propolis extracts rich in biologically active compounds [14–16]. New methods of extracting the biocompounds of propolis have been studied in order to replace the conventional ethanolic extraction method [17–20] One of the most promising methods of extraction is with supercritical fluids [21]. This method has been demonstrated to be effective for application in chemical processes, petrochemical, pharmaceutical and food processing. This method is considered to be a clean technology and has the capacity to retain the antioxidant properties of the obtained extracts through its use of low temperatures, which is an important characteristic for the pharmaceutical and food industries [21–22].

Several studies have shown the antimicrobial [23–25], anti-inflammatory [26], cytotoxic [27–28] and antiparasitic properties [29] as well as the immunomodulatory [30–31] and anti-leishmanicidal effects of different propolis extracts from different sources, including red propolis from Alagoas, and green propolis from Paraná, Bahia and Minas Gerais in Brazil [18–19]. Propolis has been widely used in several disease models, showing great potential in protective immune response against leishmaniasis [32–33]. Miranda et al. [34] showed that nitric oxide and Brazilian propolis extract combined accelerates tissue repair by modulating cell migration, cytokine production and collagen deposition in experimental leishmaniasis, highlighting a new therapeutic option that can be considered for further *in vivo* investigations as a candidate for the treatment of cutaneous leishmaniasis.

In view of these findings, it is of extreme interest for the industry to search for new technologies that ensure the preservation of extracts and their active biological compounds. Furthermore, propolis extracts are constituted by biologically active components with promising biological activities and can be investigated for the formulation of new drugs. Considering the various applications of propolis, the objective of this study was to perform the chemical characterization, evaluate the antioxidant capacity and antimicrobial activity of propolis extracts obtained by two methods of extraction (ethanolic and supercritical) from three samples (brown, green and red) collected in different geographical regions of Brazil (state of Bahia). The anti-leishmanicidal effects of the green and red propolis extracts obtained by the ethanolic extraction in the infection by *Leishmania braziliensis* were also studied.

## Materials and methods

### Obtaining and processing of propolis samples

Approximately 1000g of samples of green and brown propolis from Vitória da Conquista (Barra do Choça -14.863131, -40.552506) and red propolis samples from Canavieiras (-15.669756, -38.952456) state in Bahia, Brazil, were donated by the company Apis Jordans (S1 Table). The samples of propolis (red, green and brown) were crushed in a grinder (Philips–Brazil) in order to obtain an adequate granulometry (approximately 0.300 mm) to increase the surface area and homogenize the start material in the extraction processes. The samples were kept in a refrigerator at -20°C in bottles (small quantities 25 g) protected with laminated paper in inert atmospheric conditions (N_2_) to avoid degradation of the material.

### Characterization of raw propolis

The analysis of humidity, protein and total ash contents were performed following the official methods of the Association of Official Agricultural Chemists (AOAC) [35]. The fiber content was obtained using an automatic fiber analyzer (A-220, ANKON, New York–USA) and was based on the Van-Soest et al. [36] method. The quantification of the water activity was performed using a decagon LabMaster (Novasina, Lachen–Switzerland), with a CM-2 electrolytic cell (25°C). Total lipids were extracted and quantified using the cold extraction method described by Bligh & Dyer [37]. All of the analyses were executed in triplicate.

### Obtaining propolis extracts by conventional extraction (ethanolic)

The ethanolic extracts of propolis were made by adding 15 mL of ethanol (80%) to 2 g of crushed and homogenized propolis [38]. The extraction was performed at 70°C for 30 minutes under constant agitation in a Shaker incubator (MA 420/Marconi–Brazil), at 710-rpm. After that step, the extract was centrifuged (Centrifuge SIGMA 2–16 KL, USA) at 8800 rpm at 5°C for 10 minutes and the supernatant was transferred to glass test tubes (15x160 mm). 10 mL ethanol (80%) was added to the residue in the centrifuge tube, and the centrifugation was repeated [5,21]. All the extracts were maintained in inert atmospheric conditions (N_2_) and at a temperature of 5°C to avoid degradation.

### Obtaining propolis extracts by supercritical fluid extraction (SFE)

The extracts were obtained using a SFT-110 Supercritical Fluid Extractor (Supercritical Fluid Technologies, Inc.–USA). The equipment is composed of a high-pressure bomb (capacity of up to 10,000 psi), an extraction cell (capacity of 100 mL), an oven (containing a pre-warmer), a static/dynamics valve and restrictor valve, a flow meter, a flux totalizer (ITRÓN, ACD G1.0, Argentina) and a CO_2_ cylinder (White Martins–Brazil). A CO_2_ cylinder with a fishing tube was used to ensure that only CO_2_ in its liquid state was used in the system, a requirement of the SFT-110.

In each experiment, the extraction cell comprised 5 g of ground propolis sample with 1% ethanol as co-solvent (m/m), wool, and glass pearls. The extraction conditions were as follows: S/F, 110 (mass of CO_2[solvent]_/mass of propolis_[solute]_); co-solvent, 1.0% ethanol (m/m); pressure, 350 bar; temperature, 50°C; CO_2_ flow, 6 g·min^-1^. The extraction time was about 60 min [5,39,40].

At the end of the extraction, the vials containing the extracts were covered with aluminum foil and kept in inert atmospheric conditions (N_2_) and at a temperature of 5°C to avoid degradation of the material.

### Determination of total phenolic compounds

The analysis to determine the total phenolic compounds in the propolis extracts were performed using the spectrophotometric Folin-Ciocalteu method described by Woisky and Salatino [41], using gallic acid as a standard. Ethanol was used to dissolve the extracts in order to obtain a concentration of 0.1 mg.min^-1^. Afterwards, 0.5 mL of the aliquot of the extract was taken and mixed with 2.5 mL of aqueous Folin-Ciocalteau solution (10%) and 2.0 mL of sodium carbonate at 7.5%. The solution was placed in a thermo-regulated bath at 50°C for 5 minutes, and then the absorbance was measured in a spectrophotometer (Lambda 25 UV/vis Systems–PerkinElmer, Washington-USA) at 765 nm. The results of the concentrations of total phenolics were compared to a standard curve of gallic acid (gallic acid equivalents EGA) (mg EGA/g) under the same conditions. All the analyses were executed in triplicate.

### Determination of flavonoid content

The flavonoid content determination of the brown, green and red propolis extracts was performed using a spectrophotometer (Lambda 25 UV/vis Systems–PerkinElmer, Washington-USA) at 415 nm. The solution was prepared using aluminum chloride at 2.0% in methanol [42] in a 1:1 solution. The same procedure was performed using known solutions of quercetin standard to elaborate a standard curve. Furthermore, a blank sample was prepared under the same conditions and the quantity of flavonoid content was expressed as quercetin equivalents (EQ) (mg EQ/g). All the analyses were executed in triplicate.

### Determination of antioxidant activity (2,2-Diphenyl- 1-picrylhydrazyl–DPPH)

The evaluation of the antioxidant activity of the extracts was performed using 1,1-diphenyl-2-picrilidrazil (DPPH) according to the methodology described by Yen and Wu [43]. The extracts were diluted to five concentrations (90–190 μg.mL^-1^) in triplicates.

Afterwards, 1.0 mL of each dilution was transferred to a test tube containing 3.0 mL of ethanolic solution of DPPH (0.004%). After 30 minutes of incubation in the dark at room temperature, the reduction of the free radical DPPH was measured by reading the absorbance using a spectrophotometer (Lambda 25 UV/vis Systems–PerkinElmer, Washington-USA) at 517 nm. A blank sample was prepared using ethanol instead of the sample. Eq 1 was used to calculate the capacity to sequestrate free radical expressed as a percentage of the radical oxidation inhibition. The IC_50_ value (necessary concentration of the extract to sequestrate 50% of DPPH radical) was calculated through the line equation based on the concentrations of extracts and its respective percentages of radical DPPH sequestration.

%sequestration=100‑[(finalabsorbanceofsamplex100)/blankabsorbance] (Eq 1)

### Chromatographic analysis of the propolis extracts

Catechin (1), p-coumaric acid (2), trans-ferulic acid (3), luteolin (4) and formononetin (5) were identified and quantified from the propolis extracts. First, 10 mg.min^-1^of propolis extracts obtained in the different extraction methods were prepared and dissolved in ethanol, then placed in ultrasonic bath (TECNAL–São Paulo, Brazil) for 30 minutes. A filter of cellulose ester membrane 0.45μM (Micropore) was used to filter the samples, prior to injection on a High-Performance Liquid Chromatograph (HPLC). The chromatographic analysis were accomplish using the HPLC EZChrom Elite system, which consists of a VRW HITACHI L-2130 pump, supplied with an automatic injector and diode arrangement detector (DAD) VRW HITACHI L-2455, and a VRW HITACHI L-2300 oven. The method used to promote the chromatographic separation was adapted from Daugsch [44] and Machado et al. [39]. A LiChroCART Purospher StaR RP18-e (75 mm x 4 mm i.d.) (3 μm) column (Merck, Darmastad, Germany) was used together with a LiChroCART 4–4 LiChrospher 100RP18 (5 μm) pre-column from Merck.

HPLC was performed with an elution gradient using a mobile phase of aseptic acid 5% (aqueous phase) and methanol (organic phase) in different proportions and the total time of the experiment was 70 minutes. The volume of injection was of 10 μL and the chromatographic acquisition was defined at 290 nm (DAD). To ensure the reliability of the results, a validation was done according to the National Health Surveillance Agency (ANVISA) [45] and National Institute of Metrology, Standardization and Industrial Quality (INMETRO) [46] methodologies. This step was performed in accordance to the parameters of selectivity, linearity, precision, accuracy, detection limits and quantification limits.

### Antimicrobial activity of the propolis extracts

The Minimum Inhibitory Concentration (MIC) was used to obtain the antimicrobial activity, based on CLSI/NCCLS M7-A6 documents [47]. The strains used were *S*. *aureus* (ATCC 29213) and *E*. *coli* (ATCC 25922).

The bacterial samples, which were obtained from frozen stocks stored at -20°C, were seeded on brain heart infusion (BHI) agar and incubated in a bacterial incubator (Thermo Scientific, Massachusetts, EUA) at 37°C for 24 h and then cultured on BHI agar plates to prepare the inoculum. The initial inoculum was 1–2 x 10^5^ CFU.mL^-1^ and the extract concentration varied from 3.1 to 1600μg.mL^-1^, with the aim of determining the MIC. The tests were performed in triplicate. The analysis were defined as the minimum concentration of an extract with the capacity to inhibit bacterial growth [48].

### Ethics statement

Male BALB/c mice aged 6–8 weeks were obtained from the animal care facility at CPqGM/FIOCRUZ, located in the city of Salvador, Bahia-Brazil. All animal experimentation were conducted in accordance with the Guidelines for Animal Experimentation as established by the Brazilian Council for Animal Experimentation Control (CONCEA). The present study received approval from the local institutional review board (CEUA) (protocol: CEUA-015/2015-CPqGM/FIOCRUZ).

### Parasites

In this study, a strain of *Leishmania Viannia braziliensis* (MHOM / BR / 01 / BA788) was used. The promastigotes were cultured for seven days in Schneider’s insect medium supplemented with 10% inactive fetal bovine serum (FBS), 100 U.mL^-1^ penicillin, 100 mg.mL^-1^ streptomycin, and 2 mML-glutamine in 25 cm^2^ flasks.

### Macrophage toxicity assay

BALB/c mice femurs and tibia were used to obtain bone marrow-derived murine (BMM) cells that were cultured at 37°C under 5% CO_2_ for 7 days in RPMI medium supplemented with 20% FBS, 100 U.mL^-1^ penicillin, 100 mg.mL^-1^ streptomycin, and 2 mM L-glutamine. Thereafter, 30% of a L929 cell culture supernatant was used as a source of macrophage colony stimulating factor. After differentiation, the BMMs (10^5^ per well) were plated in 96-well plates and cultured at 37°C under 5% CO_2_ in RPMI-supplemented medium for 24 hours. The uninfected macrophages were treated with the ethanolic propolis extracts at varying concentrations (5, 10, 20, 40, 80 and 160 *μ*g.mL^-1^) at 37°C for 48 h. To finalize the procedure, the cells were reincubated for another 4 h with supplemented RPMI medium containing 10% Alamar Blue. The absorbance was then read at 570 nm and 600 nm using a spectrophotometer (SPECTRA Max 190).

### Macrophage infection

BMM monocytes were isolated as described above and 2×10^5^/cells per well were seeded in 96-well plates. Macrophages were infected (10:1) with stationary-phase *Leishmania* (V.) *braziliensis* (MHOM/BR/01/BA788) promastigotes for 24 h and the treated with varying concentrations (10, 25, 50, 75 and 100 *μ*g/mL) of green and red ethanolic propolis extracts for 48 h. The media was replaced with 0. 2 mL of supplemented Schneider medium. Cells were then cultured at 24°C for an additional five days and the number of viable parasites were determined by direct counting. Amphotericin B (0.25 *μ*g.mL^-1^) was used as a positive control.

### Statistical analysis

The program Statistica16.0 from StatSoft (Tulsa, OK, USA) was used for the statistical analysis of the results and to identify significant differences between the means. An ANOVA one-way was used to identify the differences between the concentrations of phenolic compounds, flavonoids, antioxidant activity, and the concentration of the compounds by HPLC in the extracts obtained through the two extraction methods for the three propolis samples (green, red and brown). In addition, the same test was applied to evaluate the differences between the characterization analyzes of raw propolis samples. Results are expressed as mean ± standard error of the mean (n = 3). In all statistical procedures, the level of significance was set at p<0.05.

In relation to the results of infection, GraphPad Prism Software 5.0 (GraphPad, San Diego, CA) was used for the analyses. For *in vitro* experiment using cells, the Kruskal-Wallis nonparametric test with Dunn’s posttest were used for multiple comparisons. Linear trend ad hoc analysis were used to evaluate the statistical significance between the groups, considered when p<0.05. Data are presented as the mean ± standard deviation (SD) from experiments performed in quintuplicate.

## Results and discussion

### Characterization of raw propolis samples

Analyzing the physicochemical composition of propolis is important for determining the quality of this material when it is considered for use in industrial areas, such as the food, cosmetics and pharmaceutics industries. The results of the physicochemical characterization of the three different raw propolis extracts analyzed in this study are found in Table 1. Significant differences were found in the analyses of humidity, water activity and lipids.

The brown variety showed a humidity value of 8.03%, which was slightly out of the required standards for the humidity (a maximum of 8%) [26]. The green and red propolis were demonstrated to be within the standard required.

**Table 1:** Determination of the content of humidity, water activity, total ash, raw protein, total lipids and fiber of brown, green and red propolis samples.

Sample	Humidity (%)	Water activity (%)	Total Ash (%)	Protein (%)	Lipids (%)	Fiber (%)
**Brown**	8.03±0.12^a^	0.876±0.006^a^	1.35±0.19^a^	2.49±0.08^a^	11.04±0.12^b^	70.82±5.91^a^
**Green**	6.30±0.30^b^	0.803±0.003^b^	1.44±0.10^a^	2.31±0.08^a^	8.19 ±0.64^c^	70.02±6.86^a^
**Red**	7.64±0.12^a^	0.765±0.003^c^	1.43±0.05^a^	2.12±0.09^a^	15.61±1.01^a^	68.72±2.89^a^

Values showing the same letter on the same column do not show significant difference (p>0.05) through the Tukey test at a 95% confidence level.

In relation to the water activity, the samples demonstrated a value of 0.765% for red, 0.803% for green and 0.876% for brown propolis. The values are in agreement with the results of humidity, where the samples with higher humidity showed higher water activity_._ The water activity and the humidity are the parameters that permit the determination of conservation, microbial propagation and the occurrence of chemical reactions of the products [49].

Concerning the results of total ash, the green and red propolis showed similar values compared with the brown propolis. The values found for the analysis of ash proved to be slightly lower that the values found by Machado et al. [5] for brown propolis from the state of Santa Catarina (1.73%).

The importance of the determination of total ash in propolis material were due to the possibility of commercialization in a powder form, where this analysis can identify any adulteration [50]. The samples agree with the limit established by Brazilian legislation (a maximum 5%) [51].

The protein content values found in the samples showed no significant difference (Table 1). According to Bogdanov et al. [52], the content of protein in the composition that determines quality of the sample is above 0.7%. Therefore, compared with results from this work, the propolis samples were considered quality according to the literature.

The results for the lipid analysis showed that the red variety of propolis had 15.61%, which was 47.54 and 29.27% more lipid compared to the same analysis of the brown and green varieties, respectively. These values proved to be below the values found by Machado et al [5], for red propolis (65.74%) originating from Sergipe.

The fiber content values found in the samples showed no significant difference, consistent with previous results from the literature [5].

The variation found between the samples studied and with those from other studies, including the significant differences between the samples for the humidity, water activity and lipids, can be explained by the type of propolis, the flora of the region and the period of collection [50].

### Determination of content for phenolic compounds, flavonoids and antioxidant activity of ethanolic and supercritical extraction

The results for the phenolic, flavonoid analysis and antioxidant capacity of the extracts from different samples of propolis obtained through conventional (ethanolic) methods and supercritical extraction are found in Table 2. The results showed significant differences (p>0.05) for the extracts analyzed (Table 2) when comparing the extraction method for the same sample, as well as for the extracts obtained by the same method and samples of different types.

**Table 2:** Determination of the content of total phenolics (mg EAG/g), flavonoids (mg EQ/g) and the antioxidant activity by DPPH (IC_50_) of extracts of three different samples obtained by ethanolic (EtOH) and supercritical (SFE) extraction.

Samples	Phenolic compounds (mg EAG/g)	Flavonoids (mg EQ/g)	DPPH (IC_50_)
**Brown EtOH**	249.28±0.01^a^	29.67±0.01^a^	159.74±0.03^a^
**Brown SCO**_**2**_	113.41±0.01^b^	102.02±0.01^b^	371.12±0.01^b^
**Green EtOH**	374.10±0.01^c^	131.69±0.01^c^	133.25±0.02^c^
**Green SCO**_**2**_	174.31±0.02^d^	96.86±0.01^d^	263.92±0.02^d^
**Red EtOH**	481.59±0.02^e^	186.96±0.01^e^	89.90±0.02^e^
**Red SCO**_**2**_	171.33±0.01^d^	103.30±0.09^b^	141.81±0.01^f^

EtOH–Extract obtained by ethanolic extraction; SCO_2_: Extract obtained by supercritical extraction; IC_50_: Lower values of IC_50_ indicate higher activity of radical elimination.

Statistical analysis: Values showing the same letter on the same column did not show significant difference (p>0.05) using the Tukey test at a 95% confidence level.

The variations identified among the samples were already expected, considering that propolis of different types exhibit very different chemical profiles [51–52]. Furthermore, the method of extraction and solvent can change the chemical composition of propolis extract [53]. The results found in this study confirm the influence of the type and origin of the raw material [54], as well as the extraction method [55], in the composition and characteristics of the extracts. Serra Bonvehí and Ventura [56] investigated fifteen propolis samples from various botanic and geographic origins, verifying significant differences in their contents of polyphenols, flavonoids and active components.

The main chemical classes present in propolis are flavonoids, phenolics, and aromatic compounds [57]. The content of phenolic compounds varied from 113.41±0.01 (Brown SCO_2_) to 481.59±0.02 mg EAG/g (Red EtOH), whereas the content of flavonoids varied from 29.67±0.01 (Brown EtOH) to 186.96±0.01 mg EQ/g (Red EtOH) among other samples, and the antioxidant capacity varied from 371.12±0.01 (Brown SCO_2_) to 89.90±0.02 (Red EtOH) (IC_50_).

For the major procedures analyzed, the ethanolic extraction yielded the best results. The ethanolic extraction of red propolis showed 48% more phenolic compounds compared to the brown propolis and 23.89% more than the green variety. Comparing the supercritical extractions with regards to phenolic compounds, the green propolis yielded 1.7% more compared to the red propolis and 34.9% more than the brown propolis.

The results found by Tei et al. [58], for five green propolis samples from Paraiba (Brazil) and five samples from Minas Gerais (Brazil) had 70.9% less phenolic compounds compared with the results found in this study.

Frozza et al. [59] demonstrated 68.53% less phenolic compounds in red propolis and Machado et al. [5], showed 13.61% less for brown propolis from Paraná (Brazil) extracted using the supercritical fluid extraction method.

The values identified in this study for the red and green samples appeared to be higher than the values found in the literature. These results are justified by the fact that the samples were from different origins [60].

Regarding the flavonoid analysis, the red propolis extracted by the ethanolic method indicated a difference of 84.13% more compared with the brown propolis extracted by the same method, while for the green propolis, the difference was 29.56%. Among the supercritical extracts, the total flavonoid content ranged from 1.24% (brown)– 6.23% (green) to red propolis.

The green sample tested in this present study had 64.46% more flavonoids compared with the results identify by Machado et al. [5] for green propolis originating from Minas Gerais extracted by the same method (Ethanolic extraction) and 74.17% more flavonoid compounds compared to the same sample extracted by supercritical extraction. Alencar et al. [61] also found lower values of flavonoid content for ethanolic extracts of red propolis from Sergipe.

Lower IC_50_ values indicated a higher radical scavenging activity; the brown and green propolis extracted by conventional methods demonstrated 77.68% and 48.22% less antioxidant activity when compared with the red propolis, respectively. In respect to the supercritical method, the green propolis showed 86.10% less antioxidant activity in relation to the red type. Frozza et al [59] found an IC_50_ value of 270.13 for red propolis from the northeast of Brazil, showing that the red propolis studied required less mass to inhibit 50% of DPPH radical formation.

Comparing the results presented in Table 2 in relation to the extraction method, it is possible to notice a significant difference (p>0.05) between the values for the phenolic, flavonoid and antioxidant activity (DPPH), where the ethanol extraction presented the best results between the samples and in the samples of different types. These results demonstrate the importance of the extraction method in the composition of the extract.

Similar results were observed by Zordi et al. [62], who determined that the highest concentrations of antioxidant compounds from ethanolic extracts of Italian propolis were obtained, when compared with the extracts obtained by the SFE process under different conditions and using SCO_2_. Machado et al., [5] and Silva et al. [40] also found higher values of total phenols and flavonoids in ethanolic extracts of Brazilian propolis, in relation to the supercritical extracts.

Considering the different types of processes used around the world to obtain propolis extracts, ethanol is the first choice of solvent, especially due to the affinity of its chemical characteristics with the matrix. Other solvents such as ethylic ether, water, methanol and chloroform can also be used for the extraction of specific classes of propolis constituents [62–63]. According to Biscaia et al. [64], low concentrations of flavonoid, phenolic, and antioxidant activity were shown in the extracts obtained by SFE (SO_2_) and can be explained by the fact that unwanted substances such as resin, wax and other materials that are present in propolis in high concentrations can interfere with the biological potential of the extracts. The wax and other organic wastes are removed during the process of ethanolic extraction [65].

SFE extraction is currently an alternative to conventional processes, presenting numerous advantages. Although some studies show advantages in the use of SFE to obtain ecologically clean extracts and with greater biotechnological potential [17,66,67], in this study the conventional extraction was more efficient. Most polar phenolic compounds are practically insoluble in pure CO_2_, but are sufficiently soluble in a CO_2_+ethanol mixture or in a CO_2_+ethanol+water mixture, allowing for their separation on the basis of molecular weights and polarity. Monroy et al. [55] used green propolis from southeastern Brazil to obtain extracts concentrated in phenolic compounds using supercritical carbon dioxide as an anti-solvent to selectively fractionate ethanolic and hydroalcoholic extracts of green propolis by precipitation in four separators in series.

In general, red propolis presented the best levels of antioxidant compounds, regardless of the extraction method used. Red propolis has been classified as a separate type based on its unique chemical composition, particularly rich in isoflavonoids [68]. Furthermore, ethanolic extraction was more efficient to obtain extracts with higher antioxidant capacity. Extraction with ethanol is particularly suitable to obtain dewaxed propolis extracts rich in polyphenol components [14,39].

Hatano et al. [69] also studied the red propolis (from Shandong–China). Extracts obtained by ethanolic extraction showed strong antioxidant activity. The total polyphenol content, the flavonoid content, DPPH radical scavenging activity values were 433.8 mg.g^-1^ of extract, 129.6 mg.g^-1^ of extract, and 98.8%, respectively.

#### Quantification of catechin, ferulic acid and luteolin in ethanolic and supercritical extracts by HPLC

The results regarding the quantitative analysis of catechin (polyphenol), ferulic acid/congeners (aromatic acid) and luteolin (flavonoid) are shown in Table 3. Those three compounds mentioned above were found in the ethanolic extracts of brown and green propolis at different concentrations. In the ethanolic sample of red propolis, only trans ferulic acid was found.

**Table 3:** Determination of the content of chatequin, trans ferulic acid and luteolin of red, green and brown propolis extracts obtained by ethanolic extraction (EtOH) and by SFE (SCO_2_).

Samples	Chatequin (mg/g)	Trans feluric acid (mg/g)	Lutenoin (mg/g)
**Brown EtOH**	49.39	0.10	5.24
**Brown SCO**_**2**_	<LD	<LD	<LD
**Green EtOH**	76.70	0.50	4.25
**Green SCO**_**2**_	<LD	<LD	<LD
**Red EtOH**	<LD	0.60	<LD
**Red SCO**_**2**_	<LD	<LD	<LD

EtOH–Extracts obtained by ethanolic extraction; SCO_2_ –Extracts obtained by SFE (CO_2_ as supercritical fluid); <LD: below detection levels.

None of the compounds investigated were quantified for extracts obtained by extraction with supercritical fluid and the best extraction efficiency with ethanol was also demonstrated (Table 3). It is known that extraction method influences the obtained extract, and different extracts from the same propolis sample may exhibit dissimilar properties. The yield and selectivity for some compounds are directly affected by the extraction method [70–72].

Zordi et al., [62] indicated the use of supercritical CO_2_ could be as a pre-treatment of the raw propolis to facilitate the additional extraction with ethanol. However, Machado et al., [39] showed the positive influence of the supercritical extraction to obtain two compounds in samples of Brazilian green propolis (Artepillin C and p-coumaric acid).

**Fig 1:**
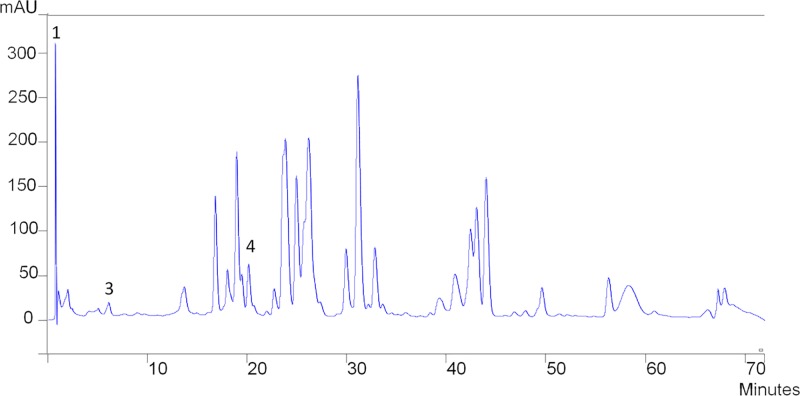
Chromatograms of green propolis ethanolic extract–(1) chatequin, (3) trans-ferulic acid and (4) luteolin.

Regarding the supercritical extraction, all of the compounds analyzed were demonstrated to be below detection levels. Fig 1 shows the chromatogram of a green propolis samples obtained by ethanolic extraction.

The content of chatequin varied from 49.39 (Brown EtOH) to 76.70 (Green EtOH) mg/g. The trans ferulic acid amount varied from 0.109 (Brown EtOH) to 0.60 (Red EtOH), whereas the luteolin compound varied from 4.25 (Green EtOH) to 5.24 (Brown EtOH).

Compared with literature data, these compounds are commonly found in different types of propolis from various regions around the world (Table 4). Our results scientifically prove that the chemical composition (biological activities) of the different propolis around the world depends on the geoclimatic conditions and the botanical source of substrate (exudates/ pollen) that bees use for production of this material, differentiating the types of propolis and their chemical characteristics. The concentration of flavonoids, phenolic compounds, aromatics and even compounds not yet determined by the scientific community determine the characteristics of different propolis samples (Table 4). The amount (mg.g^-1^ of extract) observed in this study correlates with values observed in the literature, and the different concentration, presence or not of a compound in the propolis extracts (of different regions) reinforces the previously explained results.

**Table 4:** Bibliographical review of recent studies on the quantification of the compounds catechin, ferric acid and luteolin in extracts of propolis from different regions.

Literature	Propolis	Origin	Chatequin	Trans ferulic acid	Luteolin	Method
Righi et al., [68]	Green/ Black	Brazil	**+**	**+**	**+**	HPLC/DAD/ESI/MS
Fernandes-Silva et al., [73]	Green	Brazil	**-**	**+**	**-**	CG-MS
Mendonça et al., [74]	Red	Brazil	**+**	**+**	**-**	HPLC/ LC-Orbitrap-FTMS
Hatano et al., [69]	Red	China	**-**	**+**	**-**	HPLC/PDA
Cao et al., [75]	-	China	**-**	**+**	**+**	Capillary electrophoresis system
Cui-ping et al., [76]	-	China	**+**	**-**	**-**	HPLC
Yang et al., [77]	-	China	**-**	**-**	**+**	LC-DAD
Hegazi and El Hady [78]	-	Egypt	**-**	**+**	**-**	GC/MS
Mohdaly et al., [79]	-	Egypt	**+**	**+**	**+**	HPLC
Kasiotis et al., [80]	-	Greece	**+**	**+**	**+**	HPLC-MS
Croci et al., [81]	-	Israel and Romenia	**-**	**+**	**-**	HPLC/DAD
Popova et al., [82]	-	Poland	**-**	**+**	**-**	GC/MS
Doganli [83]	-	Turkey	**+**	**+**	**+**	UPLC- ESI_MS/MS

Positive sign (+) means that the compound has been identified by the respective authors. Negative sign (-) means that the compound has not been identified by the respective authors.

### Analysis of the antimicrobial activity of extracts

The results of the MIC determination for the different (EtOH and SCO_2_) extracts of propolis (brown, green and red) tested are found in the Table 5.

**Table 5:** Determination of MIC (μg.mL^-1^) of the extracts from different samples of propolis obtained by ethanolic extraction (EtOH) and by supercritical extraction (SCO_2_).

Samples	*Staphylococcus aureus* ATCC 29213	*Escherichia coli* ATCC 25922
**Brown EtOH**	800–400	1600–800
**Brown SCO**_**2**_	1600–800	1600
**Green EtOH**	400–200	1600–400
**Green SCO**_**2**_	800–400	1600
**Red EtOH**	200	400
**Red SCO**_**2**_	400	800

The extracts demonstrated activity against the Gram-positive bacteria *S*. *aureus* (ATCC 29213) and the Gram-negative bacteria *E*. *coli* (ATCC 25922). *S*. *aureus* is a bacterium found in the skin of approximately 15% of human beings and is responsible for generating infections and food contamination. *E*. *coli* is a bacterium that lives naturally in the gut of humans and some animals but in large amounts can cause problems such as intestinal and urinary tract infections, especially in individuals consuming contaminated food or water [84].

The antimicrobial activity of the propolis were higher against Gram-positive bacteria because of the flavonoids and aromatic compounds. These chemical compounds supposedly act on the structure of Gram-positive bacterial cell walls, but the mechanism of this action is still unknown [85–86].

Scientists believe that the reason for propolis showing lower antimicrobial activity against Gram-negative bacteria is because of the multi-layered structure and higher fat content of the cell wall, which may be more resistant to propolis extracts [84,87–89].

Comparing the extraction method, the ethanolic extracts demonstrated a better antimicrobial activity compared to the supercritical extracts. The ethanolic extracts also demonstrated a higher content of total phenolic acids and flavonoids as well as better antioxidant activity. Jug et al. [90] evaluated the antibacterial and antifungal efficiency of propolis extracts obtained by different extraction methods and determined that the ethanolic extract had the best antimicrobial potential.

The red propolis appeared to have the best antimicrobial activity *in vitro* for the two bacterial strains tested, compared with the brown or green propolis. The red extract also showed the highest value of phenolic and flavonoid compounds, which may be associated with the better antimicrobial activity showed by this extract.

The extracts from the different samples tested exhibited a higher activity against Gram-positive bacteria instead of Gram-negative bacteria, which showed resistance to propolis extract, as expected [90–93]. Alencar et al. [61] demonstrated the antimicrobial activity of ethanolic and chloroform extracts of Brazilian red propolis from Alagoas state, against *S*. *aureus* ATCC 25923 (with a MIC of 50–100 EtOH extract and a MIC of 200–400) and *Staphylococcus mutans* UA159.

Studies show that antimicrobial activity occurs due to the complex synergistic effects between phenolic acids and flavonoids compounds as well as their derivatives, which are all present in propolis [94–96].

The determination of the MIC was relevant for appraising the quality of the extracts and products based on propolis [2, 97–99].

Taken together, our results showed that the red and green propolis extracts had the best values regarding phenolics, flavonoids and antioxidant capacity, as well as antimicrobial activity.

### Effects of propolis extract on murine macrophages infected with *Leishmania in vitro*

**Fig 2:**
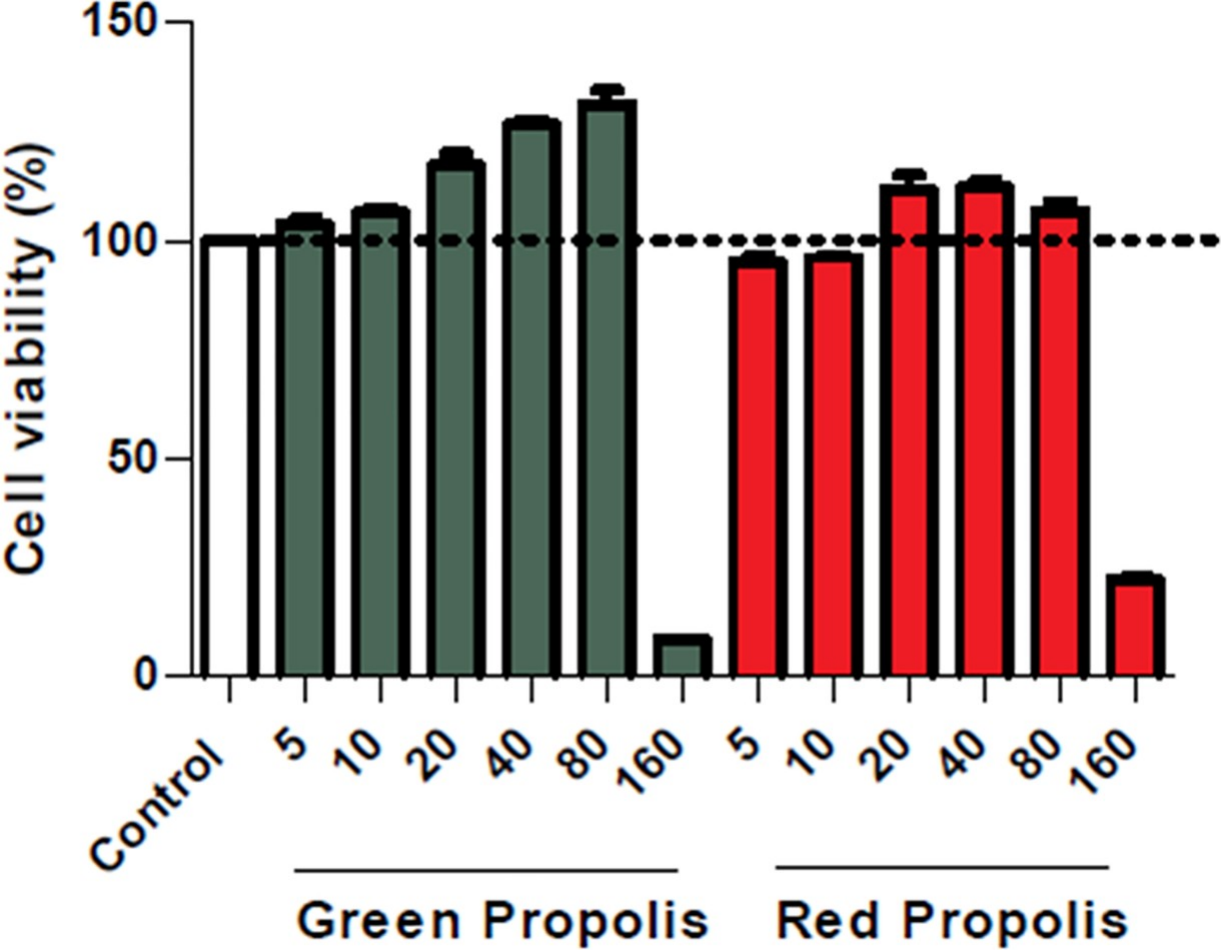
Cell cytotoxicity assessment by Alamar Blue. The data represents the viability of uninfected macrophages treated for 24 h with medium (Ctr) and the with the ethanolic extracts of propolis. The experiments were performed at least three times in quadruplicate for each experimental group. Data are shown as the mean +/- SD and are representative of three experiments.

In the next set of experiments, we tested if exposure to propolis extract reduced the intracellular viability of *Leishmania (V*.*) braziliensis*. Macrophages are the main mammalian host cell defense against *Leishmania* infection [100]. Therefore, we evaluated the macrophage viability before investigating the leishmanicidal effects of propolis extract on murine macrophages *in vitro*. According to results determined (Fig 2), the cell viability was unaffected by each concentration of the ethanolic propolis extract tested, except for the 160 *μ*g.mL^-1^ concentration, as measured by Alamar Blue assay. This finding agrees with previous reports obtained using different propolis extracts [33].

The treatment for leishmaniasis disease can be hard and painful, such that many patients give up on the treatment. In order to find an alternative path for the treatment of that disease, researchers are using natural products to fight these parasites. Some studies on the *in vitro* bioactivity of propolis have been performed against *Leishmania* species. Propolis from different geographical origins, and types (brown, green and red), have already demonstrated activity against the *L*. *amazonensis*, *L*. *braziliensis*, *L*. *infantum* and *L*. *major* [101–109].

**Fig 3:**
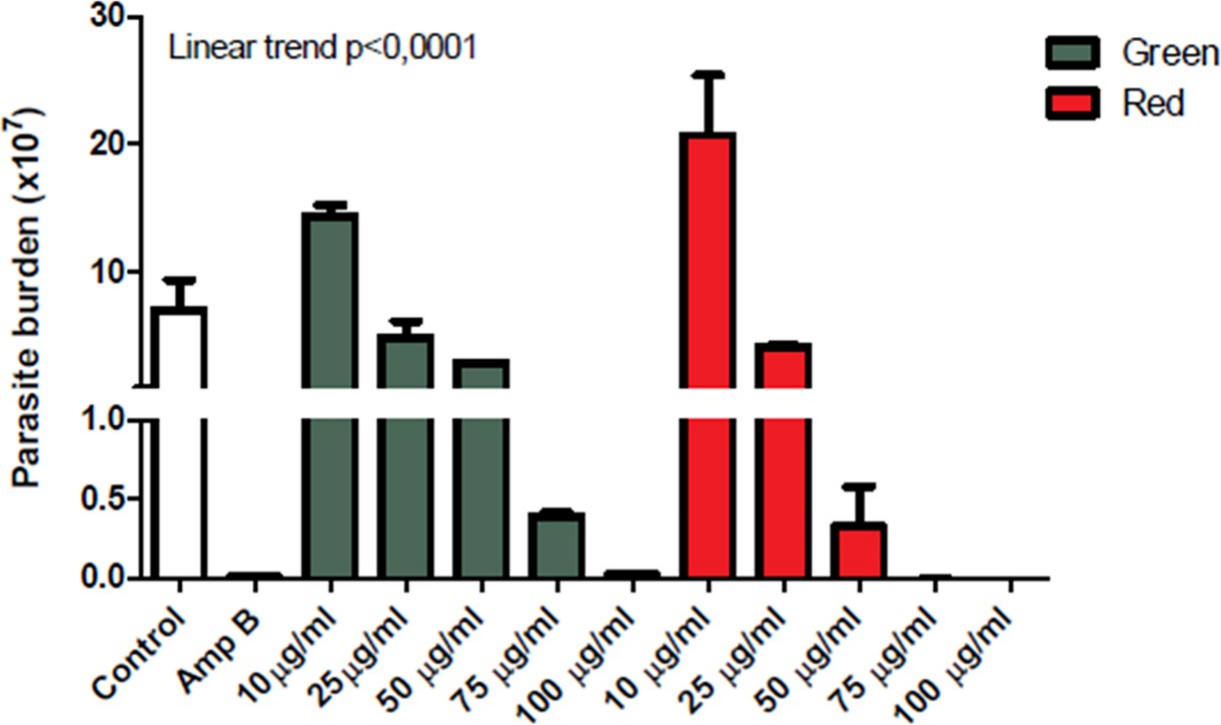
Reduction of the viability *L*. *braziliensis* promastigotes after treatment with extracts. Parasites were incubated with media alone or with ethanolic propolis extracts for 5 days. The viable parasites were counted daily with a Neubauer Chamber. The experiments were performed in quadruplicate for each experimental group (*p <0.05 and **p <0.01). Data are shown as mean +/- SD and are representative of two experiments.

The leishmanicidal effect of the two (green and red) ethanolic extracts of propolis were measured in BALB/c macrophages infected with *L*. *(V*.*) braziliensis*. All propolis extracts were demonstrated to reduce *L*. *(V) braziliensis* burden in a dose-dependent manner (Fig 3). Rebouças-Silva et al. [33] demonstrated similar results with green propolis obtained from three different pharmaceutical preparations: dry, alcoholic, and glycolic extracts.

The red propolis extract demonstrated a better reduction when compared to the green extract, corroborating the results that red propolis had more effective antioxidant activity (Table 2). The 100 *μ*g.mL^-1^ concentration showed almost the same effect as Amphotericin b, which was used as positive control. These results show that the red propolis from Bahia can be very cytotoxic against *L*. *(V) braziliensis*. Similar results were obtained by Santana et al. [69] with brown hydroalcoholic extract of propolis from the semi-arid region of Piauí (Brazil). In another study with Cuban red propolis, the antiprotozoal property was evaluated and can be associated with the chemical composition. The samples showed 3.3–16.1 μg.mL^-1^ against *L*. *infantum* [106].

Regueira-Neto et al. [104] evaluated the antileishmanial and cytotoxic activities of hydroethanolic red propolis samples collected from different Brazilian states (Pernambuco and Alagoas) and seasons whilst searching for possible activity differences. All extracts showed antileishmanial and cytotoxic activity. The propolis sample collected in Pernambuco during the rainy season showed to be more cytotoxic against protozoan parasites (*L*. *(V*.*) braziliensis* and *L*. *infantum*) and fibroblast cells and killed the parasites with lower concentrations than the sample collected in the dry season.

Ayres et al. [102] evaluated the effect of Brazilian red propolis gel (propain) alone or combined with glucantime on *L*. *amazonensis* infection. The red propolis containing high concentration of bioactive compounds (prenylated and benzophenones) showed to be the most active extract against *L*. *amazonensis*. Ethanolic extracts of propolis were capable to reduce parasite load as monitored by the percentage of infected macrophages and the number of intracellular parasites. The parasite load of macrophages was reduced by the extract (25 μg.mL^-1^), presenting no direct toxic effects on promastigotes and extracellular amastigotes.

Several biological properties of propolis extracts have been widely investigated, and its antimicrobial activity was the most studied one. Besides, its anti-inflammatory, antitumor, antioxidant, antiprotozoal, immunomodulatory, radioprotective, regenerative (tissues), healing and anti-ulcerative activities and others have been investigated as well. Here, some properties were discussed.

The results of this work confirmed the antioxidant, antimicrobial and antiprotozoal properties of propolis extracts of various types collected in different regions in the state of Bahia (Northeastern Brazil).

It was also shown that the extraction method may influence the extraction of compounds present in propolis, and, consequently, in the biological activity of the extracts.

## Conclusions

The wide diversity of the components present in raw material is evidenced by the characteristics of the raw material and the propolis extracts, and also by the compounds values obtained from two different methods. The results identified significant differences among the samples (p>0.05), which are in conformity with their place of origin. Ethanolic extraction was demonstrated to be the most efficient method for obtaining extracts with a high content of antioxidant compounds, such as phenolic and flavonoid compounds, which are associated with the biological potential of the propolis extract. Complex natural products such as propolis can result in different products, depending on the method used. Therefore, the viability of the process is related to the process yield and the product (extract) quality, in order to enhance the biological potential present in the raw material. Then, considering the advantages to achieve the concentration of biological active substances, the ethanolic extraction was best. Amongst the samples evaluated, the red propolis showed the higher biological potential, as well as the larger content of antioxidant compounds. These samples contained different constituents that may exert antimicrobial and antiprotozoal effects, which may be useful to the development of new drugs.

## Supporting information

S1 Table Identification of propolis samples from different regions of Bahia in Brazil analyzed in this study. (DOCX)

## References

[1] Bankova V. Chemical diversity of propolis and the problem of standardization. J Ethnopharmacol. 2005; 100(1–2): 114–117. 10.1016/j.jep.2005.05.004 15993016 10.1016/j.jep.2005.05.004

[2] Park YK, Alencar SM, Aguiar CL. Botanical Origin and Chemical Composition of Brazilian Propolis. J. Agric. Food Chem. 2002; 50(9): 2502–2506. 10.1021/jf011432b 11958612 10.1021/jf011432b

[3] Trusheva B, Popova M, Bankova V, Simova S, Marcucci, Miorin PL, et al Bioactive Constituents of Brazilian Red Propolis. Evid Based Complement Alternat Med. 2006; 3(2): 249–254. 10.1093/ecam/nel006 . 10.1093/ecam/nel006 PMC1475931 16786055

[4] Sena-Lopes Â, Bezerra FSB, das Neves RN, de Pinho RB, de Oliveira Silva MT, Savegnago L, et al Chemical composition, immunostimulatory, cytotoxic and antiparasitic activities of the essential oil from Brazilian red propolis. PLoS One. 2018;13(2):1–16. 10.1371/journal.pone.0191797 PMC5794096 29390009

[5] Machado BAS, Silva RPD, Barreto GDA, Costa SS, Silva DFD, Brandão HN, et al (2016) Chemical Composition and Biological Activity of Extracts Obtained by Supercritical Extraction and Ethanolic Extraction of Brown, Green and Red Propolis Derived from Different Geographic Regions in Brazil. PLoS ONE 11(1): e0145954 10.1371/journal.pone.0145954 10.1371/journal.pone.0145954 PMC4706314 26745799

[6] Daugsch A. The red propolis of northeast Brazil and its chemical and biological characteristics. D.Sc. Thesis, State University of Campinas. 2007. Available: http://www.bibliotecadigital.unicamp.br/document/?code=vtls000406573.

[7] Franchi GC, Moraes CS, Toreti VC, Daugsch A, Nowill AE, Park YK. Comparison of effects of the ethanolic extracts of brazilian propolis on human leukemic cells as assessed with the MTT assay. Evidence-based Complement Altern Med. 2012;2012 10.1155/2012/918956 PMC3182072 21966298

[8] Piccinelli AL, Lotti C, Campone L, Cuesta-Rubio O, Campo Fernandez M, Rastrelli L. Cuban and Brazilian red propolis: botanical origin and comparative analysis by high-performance liquid chromatography-photodiode array detection/electrospray ionization tandem mass spectrometry. J Agric Food Chem. 2011; 22; 59(12):6484–91. 10.1021/jf201280z 21598949 10.1021/jf201280z

[9] Simone-Finstrom M, Spivak M. Propolis and bee health: the natural history and significance of resin use by honey bees. Apidologie. 2010;41(3):295–311

[10] Abu-Mellal A, Koolaji N, Duke RK, Tran VH, Duke CC. Prenylated cinnamate and stilbenes from Kangaroo Island propolis and their antioxidant activity. Phytochemistry. 2012; 77:251–9 10.1016/j.phytochem.2012.01.012 22321386 10.1016/j.phytochem.2012.01.012

[11] Bankova V, Castro S De, Marcucci M. Propolis: recent advances in chemistry and plant origin Review article Propolis: recent advances in chemistry and plant origin. Apidologie. 2000;31(1):3–15

[12] Salatino A, Teixeira ÉW, Negri G, Message D. Origin and chemical variation of Brazilian propolis. Evidence-based Complement Altern Med. 2005;2(1):33–8 10.1093/ecam/neh060 PMC1062153 15841276

[13] Tagliacollo VA, Orsi R de O. Quality of propolis commercialized in the informal market. Ciência e Tecnol Aliment. 2011;31(3):752–7

[14] Pietta PG, Gardana C, Pietta AM. Analytical methods for quality control of propolis. Fitoterapia. 2002 73: 7–20 10.1016/s0367-326x(02)00186-7 12495705

[15] Trusheva B, Trunkova D, Bankova V. Different extraction methods of biologically active components from propolis; a preliminary study. Chem Cent J. 2007;1(1):1–4 17880743 10.1186/1752-153X-1-13 PMC1994058

[16] Sforcin JM. Biological Properties and Therapeutic Applications of Propolis. Phytother Res. 2016;30(2):894–905. 26988443 10.1002/ptr.5605

[17] Catchpole OJ, Grey JB, Mitchell KA, Lan JS. Supercritical antisolvent fractionation of propolis tincture. J Supercrit Fluids. 2004;29(1–2):97–106.

[18] Paviani LC, Saito E, Dariva C, Marcucci MC, S??nchez-Camargo AP, Cabral FA. Supercritical CO 2 extraction of raw propolis and its dry ethanolic extract. Brazilian J Chem Eng. 2012;29(2):243–51

[19] Cao J, Peng LQ, Du LJ, Zhang QD, Xu JJ. Ultrasound-assisted ionic liquid-based micellar extraction combined with microcrystalline cellulose as sorbent in dispersive microextraction for the determination of phenolic compounds in propolis. Anal Chim Acta [Internet]. 2017;963:24–32. Available from: 10.1016/j.aca.2017.01.063 28335972

[20] Pellati F, Prencipe FP, Bertelli D, Benvenuti S. An efficient chemical analysis of phenolic acids and flavonoids in raw propolis by microwave-assisted extraction combined with high-performance liquid chromatography using the fused-core technology. J Pharm Biomed Anal [Internet]. 2013;81–82:126–32. Available from: 10.1016/j.jpba.2013.04.003 23644907

[21] Machado BAS, Pereira CG, Nunes SB, Padilha FF, Umsza-Guez MA. Supercritical Fluid Extraction Using CO 2: Main Applications and Future Perspectives. Sep Sci Technol. 2013;48(18):2741–60

[22] Bashipour F, Ghoreishi SM. Response surface optimization of supercritical CO2extraction of α-tocopherol from gel and skin of Aloe vera and almond leaves. Vol. 95, Journal of Supercritical Fluids. Elsevier B.V.; 2014 348–354 p. Available from: 10.1016/j.supflu.2014.09.034

[23] Nedji N, Loucif-Ayad W. Antimicrobial activity of Algerian propolis in foodborne pathogens and its quantitative chemical composition. Asian Pacific J Trop Dis [Internet]. 2014;4(6):433–7. Available from: 10.1016/S2222-1808(14)60601-0

[24] Picoli T, Peter CM, Hoffmann JF, Soares G, Zani JL, Vargas GDÁ, et al Caracterização química e ação antibacteriana de extrato de própolis marrom da região sul do Brasil *. 2016;38(4):365–71

[25] Campos JF, dos Santos UP, Macorini LFB, de Melo AMMF, Balestieri JBP, Paredes-Gamero EJ, et al Antimicrobial, antioxidant and cytotoxic activities of propolis from Melipona orbignyi (Hymenoptera, Apidae). Food Chem Toxicol [Internet]. 2014;65:374–80. Available from: 10.1016/j.fct.2014.01.008 24412556

[26] Paulino N, Abreu SRL, Uto Y, Koyama D, Nagasawa H, Hori H, et al Anti-inflammatory effects of a bioavailable compound, Artepillin C, in Brazilian propolis. Eur J Pharmacol. 2008;587(1–3):296–301 10.1016/j.ejphar.2008.02.067 18474366 10.1016/j.ejphar.2008.02.067

[27] Franchi GC, Moraes CS, Toreti VC, Daugsch A, Nowill AE, Park YK. Comparison of effects of the ethanolic extracts of brazilian propolis on human leukemic cells as assessed with the MTT assay. Evidence-based Complement Altern Med. 2012;2012 10.1155/2012/918956 PMC3182072 21966298

[28] Utispan K, Chitkul B, Koontongkaew S. Cytotoxic Activity of Propolis Extracts from the Stingless Bee Trigona Sirindhornae Against Primary and Metastatic Head and Neck Cancer Cell Lines. 2017;18:1051–5. doi: 10.22034/APJCP.2017.18.4.1051 28547940 10.22034/APJCP.2017.18.4.1051 PMC5494215

[29] Dantas AP, Olivieri BP, Gomes FHM, De Castro SL. Treatment of Trypanosoma cruzi-infected mice with propolis promotes changes in the immune response. J Ethnopharmacol. 2006;103(2):187–93 10.1016/j.jep.2005.07.018 16214301 10.1016/j.jep.2005.07.018

[30] Fischer G, Conceição FR, Leite FPL, Dummer LA, Vargas GD' A, Hübner SO, et al Immunomodula- tion produced by a green propolis extract on humoral and cellular responses of mice immunized with SuHV-1. Vaccine. 2007; 25(7): 1250–1256. 10.1016/j.vaccine.2006.10.005 . 17084001 10.1016/j.vaccine.2006.10.005

[31] Cheung KW, Sze DM, Chan WK, Deng RX, Tu W, Chan GCF. Brazilian green propolis and its constit- uent, Artepillin C inhibits allogeneic activated human CD4 T cells expansion and activation. J Ethno- pharmacol. 2011; 138(2): 463–471. 10.1016/j.jep.2011.09.031 . 21964192 10.1016/j.jep.2011.09.031

[32] Silva SS; Miranda MM; Costa NI; Watanabe MAE, Pavanelli WR, Felipe I, Sforcin JM C-CI. Leishmanicidal activity of brazilian propolis hydroalcoholic extract in Leishmania. Sem Ciências Biológicas e da Saúde. 2015;36(2):25–34

[33] Rebouças-Silva J, Celes FS, Lima JB, Barud HS, de Oliveira CI, Berretta AA, et al Parasite Killing of *Leishmania (V) braziliensis* by Standardized Propolis Extracts. Evidence-Based Complement Altern Med. 2017; 2017:1–14 10.1155/2017/6067172 PMC5485350 28690662

[34] Miranda MM, Panis C, Cataneo AHD, Da Silva SS, Kawakami NY, Lopes LGDF, et al Nitric oxide and Brazilian propolis combined accelerates tissue repair by modulating cell migration, cytokine production and collagen deposition in experimental leishmaniasis. PLoS One. 2015;10(5):1–19. Available from: 10.1371/journal.pone.0125101 PMC4431861 25973801

[35] Association of Official Analytical Chemists (AOAC). Official methods of analysis of AOAC Interna- tional 16th ed. Washington: AOAC International; 1997

[36] Van-Soest PJ, Wine RH. Use of detergents in analysis of fibrous feeds. In: Determination of plant cell wall constituents. J Assoc Off Anal Chem. 1967; 50: 50 Available: http://catalogo.latu.org.uy/doc_num.php?explnum_id=1418

[37] Blight EG, Dyer WJ. A Rapid Method of Total Lipid Extraction and Purification. Can J Biochem Physiol. 1959; 37(8): 911–917. 10.1139/o59-099 13671378 10.1139/o59-099

[38] Park YK, Alencar SM, Aguiar CL. Botanical origin and chemical composition of Brazilian propolis. J. Agric. Food Chem. 2002; 50(9): 2502–2506. 10.1021/jf011432b 11958612 10.1021/jf011432b

[39] Machado BAS, De Abreu Barreto G, Costa AS, Costa SS, Silva RPD, Da Silva DF, et al Determination of parameters for the supercritical extraction of antioxidant compounds from green propolis using carbon dioxide and ethanol as co-solvent. PLoS ONE. 2015;10(8): e0134489 10.1371/journal.pone.0134489 26252491 10.1371/journal.pone.0134489 PMC4529176

[40] Silva RPD, Machado BAS, Barreto GdA, Costa SS, Andrade LN, Amaral RG, et al Antioxidant, antimicrobial, antiparasitic, and cytotoxic properties of various Brazilian propolis extracts. PLoS ONE. 2017;12(3):e0172585 https://doiorg/10.1371/journal.pone.0172585 28358806 10.1371/journal.pone.0172585 PMC5373518

[41] Woisky RG, Salatino A. Analysis of propolis: Some parameters and procedures for chemical quality control. J Apic Res. 1998;37(2):99–105.

[42] Marcucci MC, Ferreres F, García-Viguera C, Bankova VS, De Castro SL, Dantas AP, Valente PHM, Paulino N 2001 Phenolic compounds from Brazilian propolis with pharmacological activities. J Ethnopharmacol 74: 105–112. 11167028 10.1016/s0378-8741(00)00326-3

[43] Yen GC, Wu JY. Antioxidant and radical scavenging properties of extracts from Ganoderma tsugae. Food Chem. 1999;65(3):375–9.

[44] Daugsch A. The red propolis of northeast Brazil and its chemical and biological characteristics. D.Sc. Thesis, State University of Campinas. 2007. Available: http://www.bibliotecadigital.unicamp.br/document/?code=vtls000406573

[45] Brazil. Ministry of Health. National Health Surveillance Agency (ANVISA). Resolution n° 899, of May 29, 2003: Guide to the validation of analytical and bioanalytical methods. Available: http://portal.anvisa.gov.br/wps/wcm/connect/4983b0004745975da005f43fbc4c6735/RE_899_2003_Determina+a+publica%C3%A7%C3%A3o+do+Guia+para+valida%C3%A7%C3%A3o+de+m%C3%A9todos+anal%C3%ADticos+e+bioanal%C3%ADticos.pdf?MOD=AJPERES.

[46] National Institute of Metrology, Standardization and Industrial Quality (INMETRO). Guidelines for Chemical Testing Methods Validation. 2011. Available: http://www.inmetro.gov.br/Sidoq/Arquivos/Cgcre/DOQ/DOQ-Cgcre-8_04.pdf

[47] NCCLS. National Committee for Clinical Laboratory Standards Methods for dilution antimicrobial sus- ceptibility tests for bacteria that grow aerobically (M100-S10 (M7)). Approved standard. 5^a^ed. Wayne, PA: NCCLS; 2000

[48] Koo H, Rosalen PL, Cury JA, Ambrosano GMB, Murata RM, Yatsuda R, et al Effect of a New Variety of *Apis mellifera* Propolis on Mutants Streptococci. Curr Microbiol. 2000; 41(3): 192–196. 10.1007/s0028400101170 10915206 10.1007/s0028400101170

[49] Lewicki PP. Water as the determinant of food engineering properties. A review. J Food Eng. 2004; 61 (4): 483–495. 10.1016/S0260-8774(03)00219-X

[50] Park YK, Alencar SM AC. Botanical Origin and Chemical Composition of Brazilian Propolis. J Agric Food Chem. 2002; 50:2502–6 11958612 10.1021/jf011432b

[51] Kujumgiev A, Tsvetkova I, Serkedjieva Y, Bankova V, Christov R, Popov S. Antibacterial, antifungal and antiviral activity of propolis of different geographic origin. J Ethnopharmacol. 1999;64(3):235–40 10363838 10.1016/s0378-8741(98)00131-7

[52] Brazil. Ministry of Health. National Health Surveillance Agency (ANVISA). Normative Instruction n° 3, of January 19, 2001: Technical Regulations of identify and Quality of bee venom, royal Bee, Jelly Wax, Lyophilized Royal Jelly, Bee Pollen, Propolis and Propolis Extract. Available: http://extranet.agricultura.gov.br/sislegis-consulta/consultarLegislacao.do? operacao=visualizar&id=1798.

[53] De Lima GG, De Souza RO, Bozzi AD, Poplawska MA, Devine DM, Nugent MJD. Extraction Method Plays Critical Role in Antibacterial Activity of Propolis-Loaded Hydrogels. J Pharm Sci. 2016;105(3):1248–57. Available from: 10.1016/j.xphs.2015.12.027 26886307

[54] Toreti VC, Sato HH, Pastore GM, Park YK. Recent progress of propolis for its biological and chemical compositions and its botanical origin. Evidence-based Complement Altern Med. 2013;2013 10.1155/2013/697390 PMC3657397 23737843

[55] Monroy YM, Rodrigues RAF, Rodrigues MVN, Cabral FA. Fractionation of ethanolic and hydroalcoholic extracts of green propolis using supercritical carbon dioxide as an anti-solvent to obtain artepillin rich-extract. J Supercrit Fluids [Internet]. 2018;138:167–73. Available from: 10.1016/j.supflu.2018.04.016

[56] Serra Bonvehi J, Ventura Coll F. Study on propolis quality from China and Uruguay. Zeitschrift fur Naturforsch—Sect C J Biosci. 2000;55(9–10):778–84 10.1515/znc-2000-9-1017 11098830

[57] Escriche I, Juan-Borrás M. Standardizing the analysis of phenolic profile in propolis. Food Res Int. 2018;106(11 2017):834–41 10.1016/j.foodres.2018.01.055 29579994 10.1016/j.foodres.2018.01.055

[58] Tei ANA, Park YK, Fort P, Moraes CM, Ishiyama K. Quantificação De Cera, Compostos Fenólicos Totais E Determinação Da Atividade Anti-Radical Da Própolis Bruta. In: Resumo XVI Congresso Interno–São Paulo, Unicamp, 2008. Available: https://www.prp.unicamp.br/pibic/congressos/xvicongresso/paineis/042040.pdf.

[59] Frozza COS, Garcia CSC, Gambato G, Souza MDO, Salvador M, Moura S et al Chemical characterization, antioxidant and cytotoxic activities of Brazilian red propolis. Food Chem Toxicol. 2013; 52:137–42. 10.1016/j.fct.2012.11.013 23174518 10.1016/j.fct.2012.11.013

[60] Bankova V. Chemical diversity of propolis and the problem of standardization. J Ethnopharmacol. 2005;100(1–2):114–7. 10.1016/j.jep.2005.05.004 15993016 10.1016/j.jep.2005.05.004

[61] Alencar SM, Oldoni TLC, Castro ML, Cabral ISR, Costa-Neto CM, Cury JA, et al Chemical composition and biological activity of a new type of Brazilian propolis: Red propolis. J Ethnopharmacol. 2007;113(2):278–83 10.1016/j.jep.2007.06.005 17656055 10.1016/j.jep.2007.06.005

[62] De Zordi N, Cortesi A, Kikic I, Moneghini M, Solinas D, Innocenti G, et al The supercritical carbon dioxide extraction of polyphenols from Propolis: A central composite design approach. J Supercrit Fluids. 2014; 95:491–8. 10.1016/j.supflu.2014.10.006

[63] Marcucci MC, Rodriguez J, Ferreres F, Bankova V, Groto R, Popov S. Chemical composition of Brazilian propolis from Sao Paulo State. Zeitschrift fur Naturforsch—Sect C J Biosci. 1998;53(1–2):8–10

[64] Biscaia D, Ferreira SRS. Propolis extracts obtained by low pressure methods and supercritical fluid extraction. J Supercrit Fluids. 2009; 51: 17–23. 10.1016/j.supflu.2009.07.011

[65] Kalogeropoulos N, Konteles SJ, Troullidou E, Mourtzinos I, Karathanos VT. Chemical composition, antioxidant activity and antimicrobial properties of propolis extracts from Greece and Cyprus. Food Chem. 2009; 116(2): 452–461. 10.1016/j.foodchem.2009.02.060

[66] Wu JJ, Shen CT, Jong TT, Young CC, Yang HL, Hsu SL, et al Supercritical carbon dioxide anti-solvent process for purification of micronized propolis particulates and associated anti-cancer activity. Sep Purif Technol. 2009;70(2):190–8

[67] Chen CR, Shen CT, Wu JJ, Yang HL, Hsu SL, Chang CMJ. Precipitation of sub-micron particles of 3,5-diprenyl-4-hydroxycinnamic acid in Brazilian propolis from supercritical carbon dioxide anti-solvent solutions. J Supercrit Fluids. 2009;50(2):176–82

[68] Righi AA, Negri G, Salatino A. Comparative Chemistry of Propolis from Eight Brazilian Localities. Evidence-Based Complement Altern. 2013; 2013:14 10.1155/2013/267878 23690840 10.1155/2013/267878 PMC3639640

[69] Hatano A, nonaka T, yoshino M, ahn M-R, tazawa S, araki Y, et al Antioxidant Activity and Phenolic Constituents of Red Propolis from Shandong, China. Food Sci Technol Res. 2012;18(4):577–84

[70] Cottica SM, Sawaya ACHF, Eberlin MN, Franco SL, Zeoula LM, Visentainer JV. Antioxidant activity and composite on of propolis obtained by different methods of extraction. J. Braz. Chem. Soc. 2011; 22(5): 929–935

[71] Christov R, Trusheva B, Popova M, Bankova V, Bertrand M. Chemical composition of propolis from Canada, its antiradical activity and plant origin. Nat Prod Res. 2005; 19(7): 673–678. 10.1080/14786410512331328159 16076637

[72] Kubiliene L, Laugaliene V, Pavilonis A, Maruska A, Majiene D, Barcauskaite K, et al Alternative preparation of propolis extracts: Comparison of their composition and biological activities. BMC Complement Altern Med [Internet]. 2015;15(1):1–7. 10.1186/s12906-015-0677-5 26012348 10.1186/s12906-015-0677-5 PMC4443635

[73] Fernandes-silva CC, Salatino A, Salatino LF, Botânica D De Biociências I De, Paulo UDS, et al Chemical profiling of six samples of Brazilian propolis. Quim 11 2013;36(2):237

[74] Cristina I, Mendonça G De, Cristina I, Moraes C De, Gomes T, Souza NS De et al Brazilian red propolis: phytochemical screening, antioxidant activity and effect against cancer cells. BMC Complement Altern Med. 2015; 15:357 10.1186/s12906-015-0888-9 26467757 10.1186/s12906-015-0888-9 PMC4604764

[75] Cao YH, Wang Y, Yuan Q. Analysis of Flavonoids and Phenolic Acid in Propolis by Capillary Electrophoresis. Chromatographia. 2004;59(1–2):135–40.

[76] Zhang C, Huang S, Wen-ting W, Shun P, Xiao-ge S, Ya-jing L, et al Development of High-Performance Liquid Chromatographic for Quality and Authenticity Control of Chinese Propolis. J Food Sci. 2014;79(7):1315–22 10.1111/1750-3841.12510 24894633

[77] Yang L, Yan Q, Ma J, Wang Q, Zhang J. High Performance Liquid Chromatographic Determination of Phenolic Compounds in Propolis. Trop J Pharm. 2013; 12:771–6.

[78] Hegazi AG, Abd FK, Hady E. Egyptian Propolis: 3, Antioxidant, Antimicrobial Activities and Chemical Composition of Propolis from Reclaimed Lands. Zeitschrift für Naturforsch C A J Biosci. 2002;57(3–4):395–402. 10.1515/znc-2002-3-432 12064746

[79] Mohdaly AA, Mahmoud AA, Roby MH., Smetanska I, Ramadan MF. Phenolic Extract from Propolis and Bee Pollen: Composition, Antioxidant and Antibacterial Activities. J Food Biochem. 2015 10.1111/jfbc.12226

[80] Kasiotis KM, Anastasiadou P, Papadopoulos A, Machera K (2017) Revisiting Greek Propolis: Chromatographic Analysis and Antioxidant Activity Study. PLoS ONE 12(1): e0170077 10.1371/journal.pone.0170077 28103258 10.1371/journal.pone.0170077 PMC5245904

[81] Croci AN, Cioroiu B, Lazar D, Corciova A, Ivanescu B, Lazar MI. HPLC evaluation of phenolic and polyphenolic acids from propolis. Farmacia. 2009;57(1):52–7.

[82] Popova M, Giannopoulou E, Skalicka-Wozniak K, Graikou K, Widelski J, Bnakova V, et al Characterization and Biological Evaluation of Propolis from Poland. Molecules. 2017;22: 1159 10.3390/molecules22071159 28696397 10.3390/molecules22071159 PMC6152113

[83] Doganli GA. Phenolic Content and Antibiofilm Activity of Propolis Against Clinical MSSA Strains. Rec Nat Prod—ACG Publ. 2016;10(5):617–27.

[84] de Kraker MEA, Davey PG, Grundmann H. Mortality and Hospital Stay Associated with Resistant Staphylococcus aureus and Escherichia coli Bacteremia: Estimating the Burden of Antibiotic Resistance in Europe. PLoS Med. 2011;8(10):e1001104 10.1371/journal.pmed.1001104 22022233 10.1371/journal.pmed.1001104 PMC3191157

[85] Gonçalves GMS, Santos NP, Srebernich SM. Antioxidant and antimicrobial activities of propolis and açai (Euterpe oleracea Mart) extracts. Rev Ciênc Farm Básica Apl. 2011;32(3):349–56.

[86] Marcucci MC, Ferreres F, Guarcía-Viguera C, Bankova VS, Castro SL, Dantas AP, et al Phenolic compounds from Brazilian propolis with pharmacological activities. J Ethnopharmacol. 2001; 74(2): 105–112. 10.1016/S0378-8741(00)00326-3 . 11167028 10.1016/s0378-8741(00)00326-3

[87] Christov R, Bankova V, Tsvetkova I, Kujumgiev A, Delgado Tejera A. Antibacterial furofuran lignans from Canary Islands propolis. Vol. 70, Fitoterapia. 1999 p. 89–92. 10.1016/S0367-326X(98)00044-6

[88] Weston RJ, Mitchell KR, Allen KL. Antibacterial phenolic components of New Zealand manuka honey. Food Chem. 1999;64(3):295–301. 10.1016/S0308-8146(98)00100-9

[89] Bankova V, Boudourova-Krasteva G, Popov S, Sforcin JM, Funari SRC. Seasonal variations of the chemical composition of Brazilian propolis. Apidologie. 1998; 29(4): 361–367. 10.1051/apido:19980406

[90] Jug M, Končić MZ, Kosalec I. Modulation of antioxidant, chelating and antimicrobial activity of poplar chemo-type propolis by extraction procures. Lebenson Wiss Technol. 2014; 57(2): 530–537. 10.1016/j.lwt.2014.02.006

[91] Popova MP, Bankova VS, Bogdanov S, Tsvetkova I, Naydenski C, Marcazzan GL, et al Chemical characteristics of poplar type propolis of different geographic origin. Apidologie. 2007; 38(3): 306–311. 10.1051/apido:2007013

[92] Pires G, Pimenta F, Ribeiro L. Antimicrobial activity of two brazilian commercial propolis extracts. Brazilian J Oral Sci. 2006;5(16):967–70. doi: 10.20396/BJOS.V5I16.8641876

[93] Silici S, Kutluca S. Chemical composition and antibacterial activity of propolis collected by three different races of honeybees in the same region. J Ethnopharmacol. 2005;99(1):69–73. 10.1016/j.jep.2005.01.046 15848022 10.1016/j.jep.2005.01.046

[94] Wilson MB, Brinkman D, Spivak M, Gardner G, Cohen JD. Regional variation in composition and antimicrobial activity of US propolis against Paenibacillus larvae and Ascosphaera apis. J Invertebr Pathol. 2015; 124:44–50. 10.1016/j.jip.2014.10.005 25450740 10.1016/j.jip.2014.10.005

[95] Sawaya ACHF, Souza KS, Marcucci MC, Cunha IBS, Shimizu MT. Analysis of the composition of Brazilian propolis extracts by chromatography and evaluation of their in vitro activity against gram-positive bacteria. Brazilian J Microbiol. 2004;35(1–2):104–9. 10.1590/S1517-83822004000100017

[96] Melliou E, Stratis E, Chinou I. Volatile constituents of propolis from various regions of Greece—Antimicrobial activity. Food Chem. 2007;103(2):375–80. 10.1016/j.foodchem.2006.07.033

[97] Rufatto LC, dos Santos DA, Marinho F, Henriques JAP, Roesch Ely M, Moura S. Red propolis: Chemical composition and pharmacological activity. Asian Pac J Trop Biomed [Internet]. 2017;7(7):591–8. Available from: ttp://dx.doi.org/10.1016/j.apjtb.2017.06.009

[98] Koru O, Toksoy F, Acikel CH, Tunca YM, Baysallar M, Guclu AU, et al In vitro antimicrobial activity of propolis samples from different geographical origins against certain oral pathogens. Anaerobe. 2007; 13(3–4): 140–145. 10.1016/j.anaerobe.2007.02.001 . 17475517 10.1016/j.anaerobe.2007.02.001

[99] Kim YH, Chung HJ. The effects of Korean Propolis against foodborne pathogens and transmission electron microscopic examination. N Biotechnol. 2011; 28(6): 713–718. 10.1016/j.nbt.2010.12.006 21232643 10.1016/j.nbt.2010.12.006

[100] Liu D, Uzonna JE. The early interaction of Leishmania with macrophages and dendritic cells and its influence on the host immune response. Front Cell Infect Microbiol. 2012;2(6):1–8. 10.3389/fcimb.2012.00083 22919674 10.3389/fcimb.2012.00083 PMC3417671

[101] Santana LCLR, Carneiro SMP, Caland-Neto LB, Arcanjo DDR, Moita-Neto JM, Citó AMGL, et al Brazilian brown propolis elicits antileishmanial effect against promastigote and amastigote forms of *Leishmania amazonensis*. Nat Prod Res. 2014;28(5):340–3. 10.1080/14786419.2013.856904 24261482 10.1080/14786419.2013.856904

[102] Ayres DC, Marcucci MC, Giorgio S. Effects of Brazilian propolis on Leishmania amazonensis. Memórias do Inst Oswaldo Cruz. 2007;102(2):215–20 10.1590/s0074-02762007005000020 17426888

[103] Machado GMDC, Leon LL, De Castro SL. Activity of Brazilian and Bulgarian propolis against different species of Leishmania. Mem Inst Oswaldo Cruz. 2007;102(1):73–7 17294003 10.1590/s0074-02762007000100012

[104] Regueira-Neto M da S, Tintino SR, Rolón M, Coronal C, Vega MC, de Queiroz Balbino V, et al Antitrypanosomal, antileishmanial and cytotoxic activities of Brazilian red propolis and plant resin of Dalbergia ecastaphyllum (L) Taub. Food Chem Toxicol. 2018;119(L):215–21. Available from: 10.1016/j.fct.2018.04.029 29665415

[105] Ayres DC, Fedele TA, Marcucci MC, Giorgio S. Potential utility of hyperbaric oxygen therapy and propolis in enhancing the leishmanicidal activity of glucantimepotential utility of hyperbaric oxygen therapy and propolis in enhancing the leishmanicidal activity of glucantime. Rev Inst Med Trop Sao Paulo. 2011;53(6):329–34 22183457 10.1590/s0036-46652011000600006

[106] Monzote L, Cuesta-Rubio O, Fernandez MC, Hernandez IM, Fraga J, P??rez K, et al In vitro antimicrobial assessment of Cuban propolis extracts. Mem Inst Oswaldo Cruz. 2012;107(8):978–84 23295746 10.1590/s0074-02762012000800003

[107] Falcão SI, Vale N, Cos P, Gomes P, Freire C, Maes L, et al In vitro evaluation of portuguese propolis and floral sources for antiprotozoal, antibacterial and antifungal activity. Phyther Res. 2014;28(3):437–43 10.1002/ptr.5013 23722631

[108] Nina N, Lima B, Feresin GE, Giménez A, Salamanca Capusiri E, Schmeda-Hirschmann G. Antibacterial and leishmanicidal activity of Bolivian propolis. Lett Appl Microbiol. 2016;62(3):290–6 10.1111/lam.12543 26743801 10.1111/lam.12543

[109] Duran N, Muz M, Culha G, Duran G, Ozer B. GC-MS analysis and antileishmanial activities of two Turkish propolis types. Parasitol Res. 2011;108(1):95–105 10.1007/s00436-010-2039-z 20842509 10.1007/s00436-010-2039-z

