# Supplementary material for: Chemical characterization and biological activity of six different extracts of propolis through conventional methods and supercritical extraction
Source: PLoS One. 2018 Dec 4;13(12):e0207676. doi: 10.1371/journal.pone.0207676 (PMC6279037; doi:10.1371/journal.pone.0207676)
Supplement: S1 Table — (DOCX) [file pone.0207676.s001.docx]

**S1 Table. Identification of propolis samples from different regions of Bahia in Brazil analyzed in this study.**

| Geographic Identification | City and state of Brazil | Colour Type | Image |
| --- | --- | --- | --- |
| -14.863131, -40.552506 | Barra do Choça (Vitória da Conquista) – Bahia | Brown | 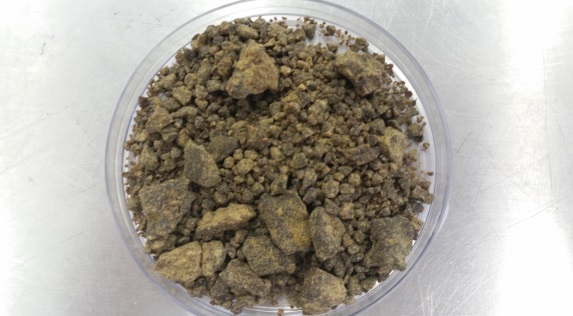 |
| -14.863131, -40.552506 | Barra do Choça (Vitória da Conquista) – Bahia | Green | 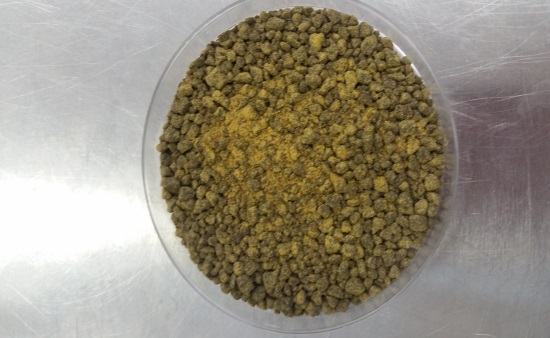 |
| -15.669756, -38.952456 | Canavieiras – Bahia | Red | 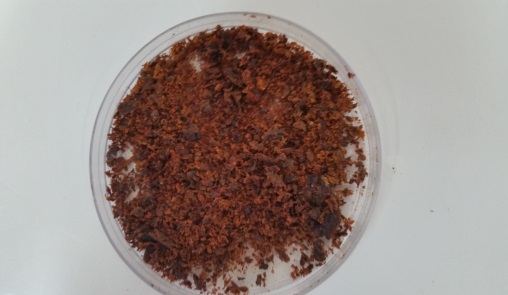 |
